# IMPACT OF AN ONLINE COURSE ON GERIATRICS AND QUALITY IMPROVEMENT SKILLS FOR NURSES IN LONG-TERM SERVICES AND SUPPORTS

**DOI:** 10.1093/geroni/igac059.319

**Published:** 2022-12-20

**Authors:** Jacqueline Telonidis, Kirstie Savage, Nanci McLeskey, Linda Edelman

**Affiliations:** University of Utah, Salt Lake City, Utah, United States; University of Utah College of Nursing, Salt Lake City, Utah, United States; University of Utah College of Nursing, Salt Lake City, Utah, United States; University of Utah College of Nursing, Salt Lake City, Utah, United States

## Abstract

Nurses working in long-term services and supports (LTSS) settings need training in geriatrics and quality improvement (QI). Our Geriatric Workforce Enhancement Program (GWEP) created an online Nurse Residency Program for nurses from other LTSS settings. Nurses (n=7) who completed the program showed statistically significant improvements in confidence in performing geriatrics skills (t = -3.12, df = 6, p = 0.01), attitudes about community-centeredness (t = -2.14, df = 6, p = 0.04), self-efficacy to engage in the treatment and assessment of depression (t = -3.06, df = 6, p = 0.01), and dementia (t = -2.04, df = 6, p = 0.04). Open-ended, satisfaction responses revealed improved self-efficacy with conducting a QI project. Empowering nurses in LTSS settings may improve quality of care, and decrease burnout and turnover. Future revisions will make the course both asynchronous and applicable to other professions in order to build stronger interprofessional LTSS teams.

